# Supplementary material for: Multi-stage resistance to Zymoseptoria tritici revealed by GWAS in an Australian bread wheat diversity panel
Source: Front Plant Sci. 2022 Oct 24;13:990915. doi: 10.3389/fpls.2022.990915 (PMC9637935; doi:10.3389/fpls.2022.990915)
Supplement: Supplementary file 1 [file DataSheet_1.pdf]

Supplementary Tables

Supplementary Table 1. Summary of the 273 accessions used in the GWAS population

Supplementary Table 2. Summary of broad-sense heritability and correlation analysis of 31 BLUEs in the study. The p values were calculated on the upper diagonal panel, while Pearson correlation coefficient values (r) were calculated in the low diagonal panel.

Supplementary Table 3. Summary of 14 QTLs discovered in the GWAS analysis

Supplementary Table 4. Summary and annotation on the candidate *R* genes detected in 10 QTL regions

Supplementary Table 5. Summary of candidate MSR QTLs published in literature

Supplementary Table 6. Summary of references reported QTL associated with *Zymoseptoria tritici* resistance

Supplementary Figures

Supplementary Figure 1. Frequency of SNP and silicoDArT markers distributed on the 21 chromosomes of bread wheat. The darker the green suggests the less the number of SNPs or silicoDArTs in the region.

Supplementary Figure 2. LD heatmap of SNP and silicoDArT markers on the 21 chromosomes of bread wheat. *r* square is used to calculate the linkage disequilibrium. Blue color indicates low linkage while red color indicates high linkage.

Supplementary Figure 3. Use of SNP and/or silicoDArT markers to calculate the average genetic distance of LD decay on A, B, D genome, and whole genome of bread wheat. The grey dotted lines represent LD is equal to 0.1, the black dotted lines represent LD is equal to 0.2. **(a)** SNP only markers were used for the decay of LD calculation; **(b)** silicoDArT only markers were used for the decay of LD calculation; **(c)** SNP and silicoDArT markers were combined for the decay of LD calculation.

Supplementary Figure 4. GWAS analysis of the traits **Plant Height (HT) and Zadoks Scale**. Manhattan and Q-Q plots for marker-trait association analysis across the whole genome of bread wheat. Orange solid dot represents the SNP marker, while dark-green solid dot represents the silicoDArT marker.

Supplementary Online data

onlinedata01_phenotype.csv

onlinedata02__silicoDArT.map

onlinedata03_SNP.map

onlinedata04_silicoDArT_genotype.csv

onlinedata05_snp_genotype.csv
